# Competency-Based Education: Developing an Advanced Competency Framework for Indonesian Pharmacists

**DOI:** 10.3389/fmed.2021.769326

**Published:** 2021-11-25

**Authors:** Sherly Meilianti, Felicity Smith, Lina Bader, Roy Himawan, Ian Bates

**Affiliations:** ^1^Department of Practice and Policy, School of Pharmacy, University College London, London, United Kingdom; ^2^International Pharmaceutical Federation, The Hague, Netherlands; ^3^Indonesian Pharmacist Association, Jakarta, Indonesia; ^4^Pharmaceutical and Medical Devices, Ministry of Health, Jakarta, Indonesia

**Keywords:** competency-based education, framework, Indonesia, pharmacist, transformation, advancing pharmacy

## Abstract

**Introduction:** Pharmacists need to be adaptable, flexible, and capable of advancing their practice to adapt to rapidly changing population health needs. We describe an educational approach to pharmacy workforce transformation in Indonesia through an advanced practice competency framework development using an “adopt and adapt” methodology.

**Methods:** The competency framework development process comprised a translation phase, an adopt and adapt phase, validation through a nationwide mapping survey, and a completion phase through leadership consensus panels. We conducted a forward-backwards translation of a previously validated Advanced to Consultancy Level Framework (ACLF) to yield the Indonesian Advanced Development Framework (IADF) draft. The subsequent adoption and adaptation process was conducted through a series of consensus panels. We validated the IADF through a nationwide workforce survey. The final phase included leadership consensus panels with the professional leadership body in Indonesia. We analyzed the qualitative data thematically and the quantitative data using a Multiple Correspondence Analysis (MCA) technique.

**Results:** We identified conceptual challenges in adopting and adapting the existing ACLF, which were addressed by providing a national glossary and concrete examples. A total of 6,212 pharmacists participated in the national workforce survey, of which 43% had <2 years of post-license (post-registration) experience. The MCA results showed that practitioner self-assessment to the IADF could discriminate their career development stages. The results also indicated a four-stage career model (including early years career training). Embedding this model in a structured national training program will enhance the professional workforce development through a more structured career journey.

**Conclusions:** We describe the first validation of an advanced competency development framework for the pharmacy workforce in a non-Anglophone country, showing the possibility of transnational applicability of this framework. We argue that this methodology can be used in Low and Middle-income countries (LMICs) for the more rapid advancement of pharmaceutical care practice.

## Introduction

Global health systems face continuous challenges, placing additional demands on the health workforce (1, 2). Development of an adaptable, flexible, accessible and capable workforce is imperative to build and maintain resilient health systems (3, 4). Health workforce development through education and training is key to preparedness and quality service delivery.

A competency-based education and training (CBET) approach has been implemented in many health disciplines to reform health workforce education and training (2, 5, 6). The introduction of CBET within the regulated health professions has been driven partly by the dissatisfaction with outcomes of more orthodox theory-based education models, and partly by the urgent imperative for a flexible and adaptable workforce that better meets changing population health needs (2). CBET has a greater emphasis on the outcomes needed for practice and encourages practitioners to engage enthusiastically with the advancement of other practitioners (6–8). This approach focuses on learner competencies, particularly the developmental progression of practitioners (6, 9). A critical component of the CBET model is the development of the competencies required for consistently safe and effective performance within the scope of professional practice. As a consequence, competency frameworks that support the requirements for professional practice are now more commonplace within the health professions (2, 10). Competency frameworks are used to allow an individualized learning process, where practitioners have opportunities to explore learning activities options for advancing their practice (2, 11–13). Studies show that the use of competency development frameworks assures consistency of practice, fosters continuing professional development and aids expertise development in pharmacy (14–16).

The establishment of competency frameworks is a crucial step toward CBET implementation (17, 18). There is evidence to support the effectiveness of competency-based approaches for workforce development, aided by competency frameworks, in pharmacy, medicine and nursing (19–21). The nurse profession designs a competency framework as “levels” to demonstrate achievement from novice to advanced practitioners (7). This concept has also been used in the pharmacy profession, such as the Global Advanced Development Framework (GADF), established by the International Pharmaceutical Federation (FIP) in 2019 (22). The GADF was originally developed from the Advanced to Consultant Level Framework (ACLF) (23). The framework comprises 34 competencies dispersed across six competency cluster areas: “expert professional practice;” “working with others;” “leadership;” “management;” “education, training and development;” and “research and evaluation.” Each competency has competency level descriptors at three levels of practice. The original design of the ACLF also included supportive evidence categories, in which practitioners could choose to support their assessment and judgment on their levels of practice to the competencies (24). There were 12 evidence categories and several supportive evidence examples within each evidence category.

Advancing the pharmacy workforce is imperative to strengthen pharmacists' role in the primary health care agenda, particularly in low and middle-income countries (LMICs), where challenges on the access to medicines expertise and primary health care are evident (25). CBET models may be considered cost-effective (26) since there is often a lack of infrastructure and resources in LMICs (25, 27). The CBET approach provides flexible delivery and tailoring of educational materials to “scope of practice” in order to specifically optimize training requirements with health needs requirements (26). Like the other LMICs, Indonesia faces similar challenges related to a need for advanced training programs to improve pharmacists' competencies in providing more comprehensive pharmaceutical services, particularly in primary healthcare settings (25, 28, 29).

A preliminary needs analysis of workforce development in Indonesia showed a need to develop a national advanced practice program and professional recognition system to support the pharmacy profession (30). A starting action to build this program was developing a framework to define advancement practice. The existing competency standard available at the national level is the Indonesian Competency standard (SKAI), which is the initial competency standard for pharmacists entering the workforce at registration (31). The scope of the SKAI is similar to the FIP Global Competency Framework, which is used to define the expected core competencies of foundation-level pharmacy practitioners (32). There was, however, no advanced competency development framework available in Indonesia at the time of this study, impeding systematic advancement of the workforce. Our study aimed to develop the Indonesian Advanced Development Framework (IADF) as a mapping and development tool for pharmacists in Indonesia to support their professional development and career progression.

## Methods

**Figure 1:**
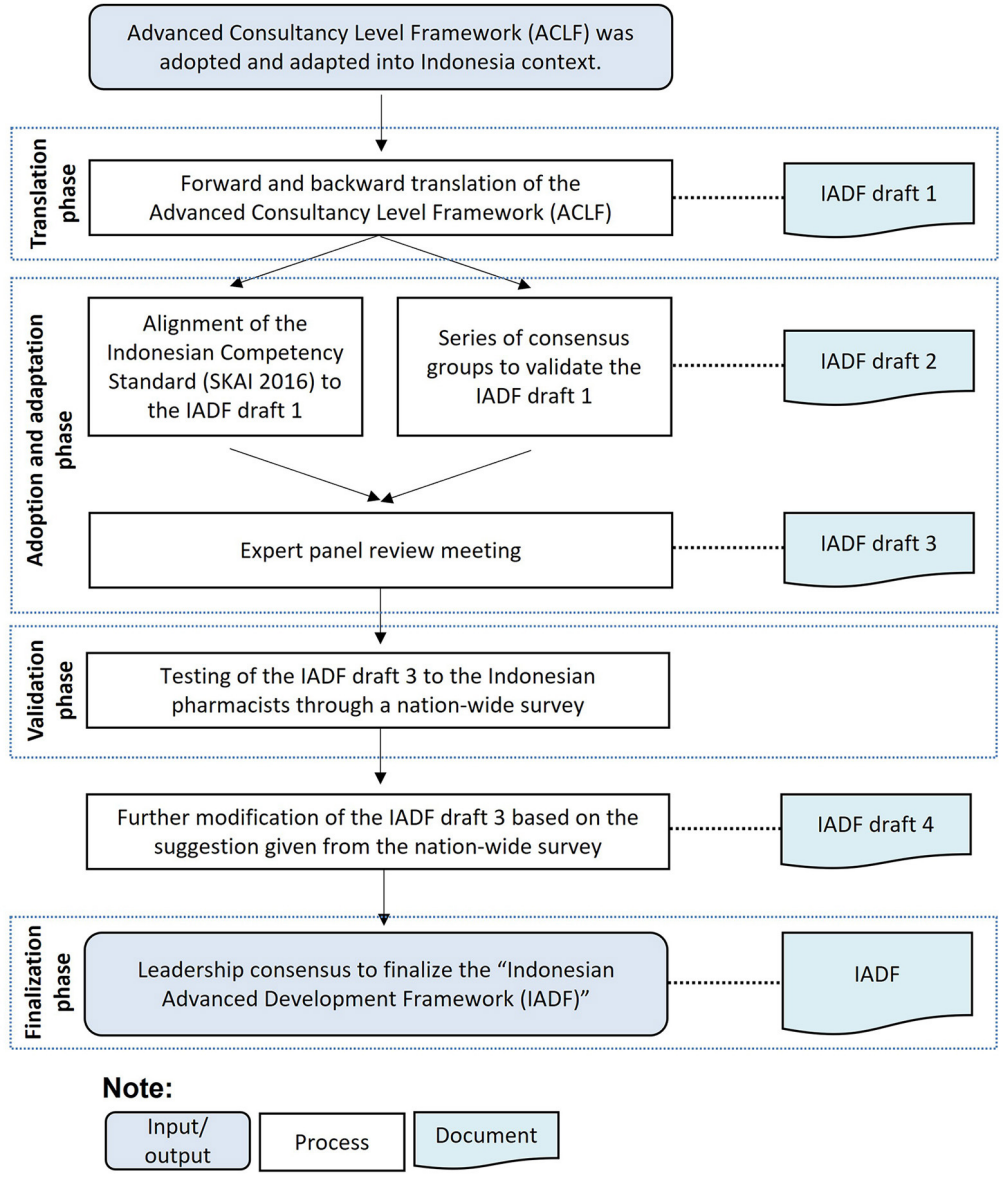
The development process of the Indonesian Advanced Development Framework.

A bottom-up approach is usually used in competency development frameworks, starting with a literature review, a series of consultation and consensus-building, nationwide stakeholder workshops or surveys with practitioners who will use the framework (33). An “adopt and adapt” approach is considered beneficial if an evidence-based framework is available, particularly in the resource-poor setting (34). The innovation described here—the “adopt and adapt” strategy—is designed to ensure sustained stewardship at national level, rather than a new national framework being perceived as an imposition originating from elsewhere. The methodological challenge was the syntactical problem of “conceptual translation” and adoption. We developed the IADF by adopting and adapting the ACLF (24), which has been used as a starting point to develop an advanced practice framework in other countries (23), including a recently validated version for generic global use published by the FIP (22). The IADF developmental process included a translation, adoption and adaptation, validation through a nationwide survey, and finalization phase through a leadership consensus panel (see Figure 1).

### Translation Phase

Two independent bilingual Indonesian pharmacists translated the ACLF to the Indonesian language (Bahasa) independently. We aided translators with a translation guideline. We organized a reconciliation meeting with translators to reach a consensus on the best wording of the IADF draft. Most decision-making in the reconciliation meeting was to modify competency statements to be read naturally in the target language. A third bilingual pharmacist conducted a back-translation of the IADF draft. The pharmacist was not familiar with the framework, mitigating for potential bias. We compared and highlighted the discrepancies between the back-translated and original versions of the IADF. A consensus on the translated version was achieved (IADF draft 1).

### Adoption and Adaptation Phase

We conducted a content analysis mapping between the existing competency regulatory standard (SKAI) and the IADF. The mapping was subsequently validated through an online discussion with the Indonesian Pharmacists Association (IAI) leadership resulting in a final mapping consensus and suggested modification to the IADF draft.

We conducted five consensus groups subsequently to validate the framework content and the supportive evidence example. We targeted practicing pharmacists working across sectors of practice and locations in Indonesia purposively and recruited them by utilizing representatives of special interest groups (SIGs) of the IAI. The participant demographic of each consensus group can be seen in Table 1. We developed and circulated consensus meetings protocols prior to the meetings. We audio-recorded the meetings subjected to participants permission. The agreed framework outcome of each consensus group was discussed in the subsequent consensus groups. The latest agreed framework and the final list of supportive evidence (IADF draft 2) were circulated to all consensus groups to gather further feedback.

**Table 1:** The demographics of participants in the consensus development groups.

**Consensus group sequence**	**Number of participants**	**Practice sectors**	**Location of practice in Indonesia**	**Years of practice**
First	16	Academic, community, government institution, hospital, industrial setting	Variety of locations	3–40 years
Second	4	Academic, community, hospital setting	East part	18–39 years
Third	6	Community, community health center, government institution, hospital setting	West part	2–16 years
Fourth	11	Community health center	Variety of locations	1–10 years
Fifth	3	Community setting	West part, central part and east part	1–14 years

We conducted expert panel review meetings to achieve consensus on the content and format of the IADF draft tool. The expert panel was purposively recruited by targeting the IAI expert panel committee (*n*: 39). This panel had valid expertise in competency development and wide experience across sectors of practice in Indonesia. The review meeting outcomes were circulated to provide feedback. Follow-up meetings with the expert panels were conducted to integrate data from the mapping of the SKAI to IADF. The consensus IADF draft was converted to an online survey platform and is shown in Supplementary Material.

### Validation Phase

We conducted a cross-sectional online survey to investigate the feasibility and validity of the IADF as a tool for self-assessment of pharmacists. The questionnaire consisted of three parts; information relating to pharmacists' background and current practice; self-assessment on current stage of practice using the framework [providing evidence to support their self-assessment—see reference (31)]; and finally, reflections on the framework's usability and feasibility through open-ended questions. The survey was circulated for 2 months through social media and to the conference delegates of the 2019 annual congress of the IAI. A reminder posting was sent every 2 weeks.

The data were coded and cleaned before analysis. The responses were analyzed descriptively using the frequency distribution of cluster staging. To calculate the aggregated staging of each cluster for each respondent (there were 6 clusters in the framework), we calculated the median value of staging within the specific cluster. We used Multiple Correspondence Analysis (MCA) to explore the relationships among the staging of the IADF's cluster, the overall staging (the 'summary' of the IADF clusters) and the practice demographic categories of the sample. The workplace categories used were: academic, community, community health center, government institution, hospital, industry, wholesaling, and others. We analyzed open-ended questions thematically to explore respondents' feedback on the feasibility of the framework for developing their practice and potential improvements.

### Finalization Phase

We prepared a briefing paper to guide discussion within the Indonesian Pharmacists Association (IAI)'s committee to finalize the IADF; survey findings were incorporated into the IADF draft. Apart from the briefing paper, a set of prompts was given to assist the committee in providing their comments. The IAI committee is a policy-making committee consisting of 64 members of the IAI's central committee covering all sectors of practice and specialisms. Recommendations from the committee were discussed with the IAI leadership to finalize the framework content.

### Ethical Consideration

An ethics approval was obtained from the UCL Research Ethics Committee (Application 11819/002). Participation in this study was voluntary, and the data were kept confidential. A cover letter was provided at the beginning of a survey link, which included consent from participants to participate. A two CPD credit from the IAI was given as an incentive for participants who completed the survey.

## Results

### Translation Phase

In general, the ACLF could be translated into the Indonesian language (Bahasa). Also, no major issue was found related to the equivalency between the original and the back-translated version. We developed a glossary to describe some terminologies that were not common in Indonesian cultures, such as “governance,” “core area,” “defined area,” “mentor,” “across boundaries,” “role model,” and “peer review.”

### Adoption and Adaptation Phase

The content analysis findings showed that all units in the SKAI were aligned with competencies within the IADF draft. The most common cluster mapped to the SKAI was the “expert professional practice,” followed by the “working with others” cluster. The least common cluster mapped was the “education, training and development,” suggesting that not many competencies within this cluster were set as a standard for initial registration. Some competencies within the IADF draft were not mapped in the SKAI, such as “leadership skill,” “supervises others undertaking research,” and “establish a research partnership.”

This alignment process signposted some modifications in the IADF draft. For example, it was stated that pharmacists should be able to decide and create a logic assessment in the SKAI, which could be mapped to stage 2 of “reasoning and judgment” competency within the IADF draft. However, stage 2 of the IADF emphasized the complexity of the situation that pharmacists manage. Also, the focus of the competency in the IADF was about working in a team and a broader context, while in the SKAI, the focus was more about personal skills. Thus, a contextual explanation was added to describe the staging within the IADF draft.

While a consensus was achieved that the IADF draft could advance the pharmacy workforce, some questions were raised during the consensus meetings. The questions raised focused more on framework implementation, such as the relationship of the job description and the staging in the framework, the impact of years of experience with staging and the impact of the staging on the relationship with other colleagues. In the first competency, “expert professional practice,” a question on how this competency facilitated various practice sectors in Indonesia raised, particularly associated with the difference in pharmacists' focus working in patient-facing roles and non-patient-facing roles. The concept of “breadth” and “depth” arose in the meetings. It was suggested that the advancement was more focused on the “depth” for the patient-facing role. In contrast, for the non-patient-facing role, the advancement focused more on the “breadth” of the area that pharmacists covered. The word “self-defined” in the first competency was further explained in the glossary to ensure the generic applicability of the framework because pharmacists could specify the area by themselves. Some additional terminologies were also added to the glossary list: “professional expertise” and “service.”

### Validation Phase

A total of 6,212 pharmacists were engaged in this survey. We excluded pharmacists who indicated <2 years of experience from the analysis because we assumed they were at the “foundation stage.” Thus, a total of 3,539 responses were included in the analysis (see Table 2). Forty per cent of respondents worked in community settings, and most respondents (78%) were females. Comparing the respondents with the available data of pharmacists in Indonesia, it can be seen that the majority of pharmacists in Indonesia (52%) also worked in the community setting. Similarly, 78% of pharmacists in Indonesia were females. Most respondents (78%) had <10 years of experience, and more than half (52%) of participants practiced in Java Island. Similarly, most pharmacists in Indonesia (60%) worked on Java island.

**Table 2:** The demographics of participants included in the analysis.

**Demographics**	**Categories**	**Respondents (%)**		Population estimation of pharmacists (%)^*^	
Sector of practice	Academic	162 (5%)	*n*: 3,539	Not recorded	*n*: 24,514
	Community	1,406 (40%)		12,714 (52%)	
	Community health center	259 (7%)		1,890 (8%)	
	Industry	176 (5%)		582 (2%)	
	Government institution	144 (4%)		362 (1%)	
	Hospital	959 (27%)		7,734 (32%)	
	Wholesaling	225 (6%)		1,232 (5%)	
	Others	208 (6%)		Not recorded	
Gender	Male	779 (22%)		13,931 (22%)	*n*: 65,104
	Female	2,760 (78%)		51,083 (78%)	
Location of practice (island)	Sumatera	849 (24%)		11,132 (16%)	*n*: 69,056
	Java	1,835 (52%)		41,487 (60%)	
	Bali and Nusa Tenggara	206 (6%)		3,329 (5%)	
	Borneo	317 (9%)		4,848 (7%)	
	Sulawesi	216 (6%)		6,146 (9%)	
	Maluku and Papua	116 (3%)		1,914 (3%)	
Years of experience	3–5 years	1,043 (30%)		No data were available
	6–10 years	1,692 (48%)		
	11–15 years	476 (13%)		
	16–20 years	191 (5%)		
	More than 20 years	137 (%)		

* *Based on the report of the Indonesian Pharmacists Association's program plan 2019–2020 using the number of pharmacists who have registered to the Pharmacy Informational System (SIAp)*.

#### Descriptive Analysis of the IADF Mapping

**Figure 2:**
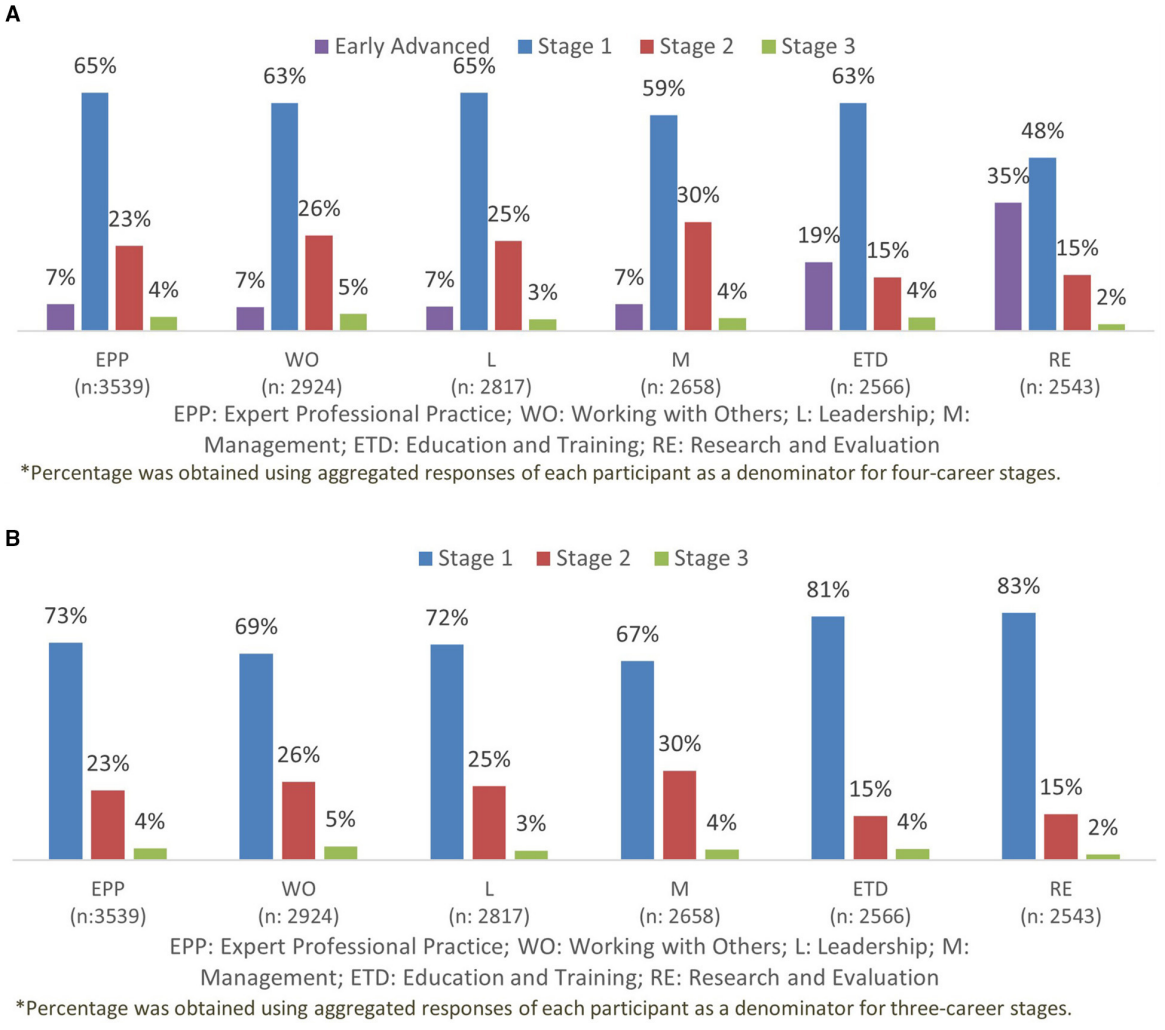
**(A)** Four career stages. **(B)** Three career stages.

There are two possible mapping distributions, or models, of workforce mapping. The first model included four sequential career stages: “early advanced,” “advanced stage 1,” “advanced stage 2,” and “advanced stage 3” (see Figure 2A). A second model describes three sequential career stages as a result of merging “early advanced” with “advanced stage 1” categories to become an enlarged “advanced stage 1” (see Figure 2B). Looking at both models, there was greater variation in the proportional distribution in the four career stages model 1 (Figure 2A) compared with the three career stages (Figure 2B). With the latter, as model 2, many pharmacists were situated within Stage 1, which was about two-thirds of the workforce. On the other hand, in model 1 (Figure 2A), there was a more balanced proportional grouping across career development stages in this surveyed sample. Another observation from Figure 2A was that the “research and evaluation” cluster has the lowest percentage than other clusters showing the workforce to be least prepared for engagement with research or evaluation activities (for example, evidence-led practice) in healthcare settings.

#### Multiple Correspondence Analysis of the IADF Mapping

**Figure 3:**
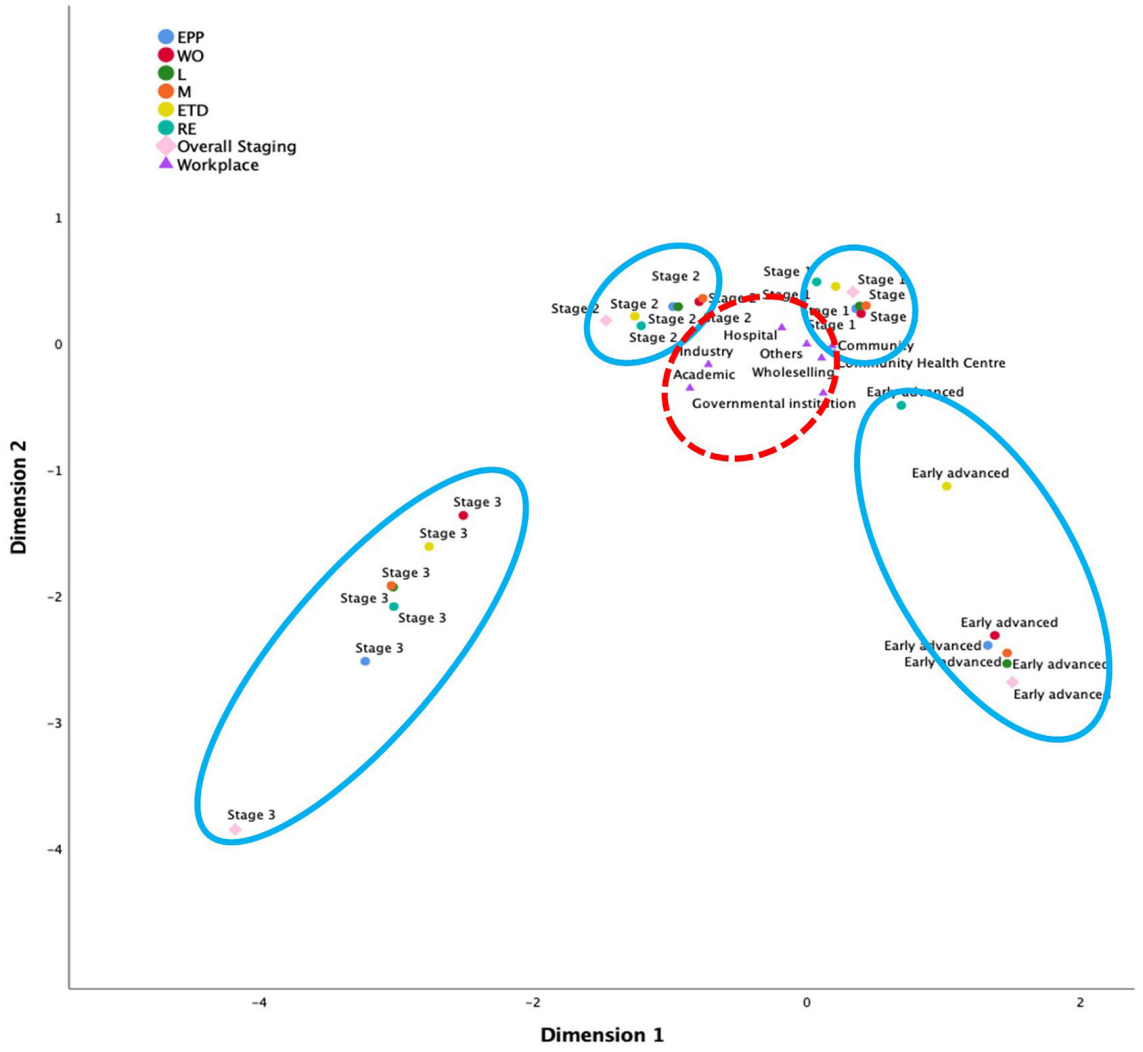
MCA using the four-stage career development pathway model; advancement stages are grouped in blue, workplace groupings are in red.

The MCA outcomes, using two dimensions, explored relationships of the “staging” of each cluster and the workplace. The Cronbach's value for each dimension was higher than the minimum acceptable threshold value of 0.5 (35). The analysis summary indicated that this model was robust for association pattern discovery. The joint category plot of MCA provided a descriptive pattern of all categories of self-assessed staging and sector for the surveyed sample (see Figure 3). The “blue” groupings showed that the self-assessed career stages were grouped and separated from each other. It showed that the IADF—when used as a self-assessment tool—was able to discriminate between career development stages in this sample. The “red” grouping showed an apparent clustering of the practice sector within the context of self-assessment with the IADF. It showed that the workplace setting did not appear to influence self-assessment toward any particular staging.

#### Thematic Analysis Findings

While participants were invited to provide any comments related to the framework improvement, there were no specific comments from participants related to the competencies or descriptors in the framework. One improvement that many participants stated was to specify the framework for each practice sector when being implemented. They also expressed a need to have specific evidence for each staging and each competency.

“It is necessary to elaborate more detailed examples of evidence, or a form of questions that are closer to the pharmacy services, and not just general questions related to national/international involvement” (participant 24, male, hospital, 1-year experience).

This suggestion did not affect the framework changes; however, it was noted for the implementation plan.

### Finalization Phase

There were no comments received from the IAI committee related to the framework content; however, three recommendations were obtained from the community health center special interest group (SIG), the industrial SIG and hospital SIG focusing on framework implementation. They highlighted a need to provide more information on the “breadth” and “depth” of “expert professional skill,” particularly related to each sector of practice. Creating a flow chart in the professional development process using this framework was suggested. They recommended including SIGs in the process of professional recognition system development. This recommendation was considered for the framework implementation plan, where each SIG will discuss this framework within their group to provide more specific examples of the supportive evidence for the framework.

## Discussion

Prior studies have noted that competency development frameworks facilitate the improvement of health practitioners' performance (19, 36). Our study provided evidence of developing a competency framework to support pharmacists working at an advanced level of practice. With a variation of practice scope in Indonesia, we aimed to develop a generic framework that demonstrates a continuum of professional development and growth from novice toward advanced practice. An important finding from our analysis showed the generic applicability of the IADF; thus, it could support professional development for a wide variety of sectors in Indonesia. This concept of generic advancement is similar to those in other professions, such as nursing (37). A systematic review conducted by Udoh et al. (18) highlighted that there were currently only four evidenced-based advanced generic competency frameworks for pharmacists implemented nationally in the United Kingdom (38), Australia (39), Singapore (40), and South Africa (41). These competency development frameworks were adopted and adapted from the same source, the FIP Global Competency Framework (42) and ACLF (24). Our study has added evidence on the development and validation process for an advanced practice framework in a non-anglophone environment and geographical region.

Our study considered all processes for competency framework development highlighted in a scoping review conducted by Batt et al. (36), with the key principle for our framework development to be direct engagement with those who will be impacted by the framework, i.e., Indonesian pharmacists, in order to maximize sustained implementation success (43). The development of competency frameworks for health professionals has primarily relied on the utilization of qualitative and consensus development processes to seek acceptance (44). Our study ensured the inclusion of key stakeholders such as the practitioners and the professional leadership body to refine and seek agreement.

CBET prioritizes the acquisition of competencies over time and allows individualization of learning that relates to personal motivation and self-regulated learning progression (45). Our study supports this concept by allowing pharmacists to self-assess themselves to the framework, identify their current stage of practice, and identify potential learning needs. It is designed to be applicable across all sectors to induce pharmacists into advanced practice progression by evaluating their competencies and self-defined expertise. This will also promote learner centredness for responsibility for personal development and pathway planning toward advancement (12). Previous studies highlighted a need to actively train practitioners in reflective practice and conduct self-assessment to identify learning needs and gaps for practitioners (46, 47). We recommended this as the next step when implementing the framework. Previous studies related to CBET in health professions highlighted that advancement is based on a demonstration of trustworthiness, a summative concept derived from diverse viewpoints (faculty, patients, peers, other professional colleagues) (6, 34). As in our study, healthcare practitioners develop a dynamic personal portfolio with sufficient framework evidence tailored to individual and healthcare environment needs (6). Our study further supports this idea where the IADF provides a basis for developing a national professional recognition system, which has been used in some countries that implemented the advanced practice framework (48–50). This system could signpost opportunities for the continued advancement of the practitioners and open up new practice and scientific development opportunities for professionals. Thus, it can provide credible evidence of the pharmacist's role in medicines expertise, patient safety, and enhancing the quality and impact of pharmaceutical care provision (11).

We identified translational and cultural challenges in developing the IADF. However, a consensus was achieved in that the IADF can be used to advance pharmacy practice in Indonesia. The findings of the most mapped clusters of the SKAI and the IADF were not surprising because the SKAI was aimed as a standard for initial registration for pharmacists. The “expert professional practice” cluster within the IADF relates to the scope of practice that pharmacists defined by themselves; thus, it is relevant to the foundational competencies defined in the SKAI. Some competencies in the IADF, which were not available in the SKAI, highlighted a progression gap between the SKAI to the IADF. These competencies were reasonable since supervising others, establishing partnerships, and developing leadership skills might be more appropriate for advanced pharmacists. These skills are developed through practice experience. This overlapping supported the evidence that the IADF was a generic framework that could be adopted and adapted to the Indonesian context. Prior studies on competency components in healthcare professions have focused on leadership skills, organization and management skills, personal and professional practice, collaborative skills, interpersonal and interprofessional communication skills and research and education; our development and outcome is consistent with this literature (17, 34, 37, 39, 41).

Our study aimed to utilize the IADF as a mapping and development tool to support career progression for Indonesian pharmacists, particularly for the advancement of pharmacists working in complex and challenging pharmaceutical healthcare environments. Our findings suggest that it is crucial to ensure that both early- and mid-career pharmacists can advance their careers with the support of a validated systematic framework. Our study sample showed significant interest from the early-career practitioner sub-sample, with 43% of respondents having fewer than 2 years of experience. We believe this is an opportunity for further research in developing structured early career foundation training. Based directly on the IADF self-assessment distribution patterns, introducing early career foundation training for the “excluded” sample of <2 years of registration experience, this proposed foundation training would act as a bridging CPD program for the current “early advanced group.” A structured foundation training (FT) program would support not only the early-career pharmacists but also those who want to practice following a career break. This finding also raises an intriguing question regarding how the Indonesian workforce will look 3–5 years after implementing the national training programs.

For the education and training providers, the IADF can also be used as a tool to map a useful and relevant education and training provision according to workforce needs. The IADF development we describe here could be a basis for National training programs (NTPs). The NTPs could focus on better management and progress with medicines management for long term conditions and non-communicable diseases in primary care settings, a particularly urgent primary health care need in Indonesia.

### Limitations

This study has limitations. The use of an online survey may have introduced a self-selection bias but balanced with greater outreach. The perceived self-assessment concept used in the study relates to the belief that participants have about themselves; therefore, there was a possibility that the confidence or situation that pharmacists had when completing the survey influenced the self-assessment that they did. This might affect the reproducibility of findings. Another limitation is that only <10% of pharmacists completing this survey. However, the number of responses received is close to the proportion of demographics profile of pharmacists in Indonesia, providing supporting evidence that our data sample is representative. Also, to the best of our knowledge, this is the largest survey for pharmacists ever conducted in Indonesia. Despite these limitations, in relation to framework development and validation, this study used an existing validated framework and utilized several methods to address the equivalency concepts between frameworks.

Our study highlighted benefits of the “adopt and adapt” general purpose methodology. The framework development process itself is more effective than building a framework from scratch. Our starting framework has been used in other countries with an established credibility and validity from testing in the source population; this reduces the amount of methodological work to validate the framework. The adoption and adaptation process, however, could be problematic if it is not done robustly as it relies on the methodological process conducted by the development team. Phrasing and wording within the framework could have contextual differences in the target population, so “re-contextualization” is critical to ensure the framework aligned with the target population. There may be also local barriers to facilitation when implementing the framework, therefore the bottom-up approach conducted in this study ensures greater ownership by the Indonesian workforce.

## Conclusion

Our study described the initial steps of advancing Indonesian pharmacists by developing a valid and consensus driven advanced practice competency development framework, the IADF. Further research might explore this framework's implementation in a holistic national CBET program to assess the impact. Our study also provided evidence on a top-down and bottom-up approach in developing policy for advancing Indonesian pharmacists. To our knowledge, this framework validation process was the first validation of an advanced competency framework in a non-anglophone LMIC. Our study signposted possibilities for transnational collaboration in developing a country-level advanced framework to accelerate country progress on pharmacy workforce education and training development.

## Data Availability Statement

The anonymized raw data supporting the conclusions of this article will be made available by the authors, without undue reservation.

## Ethics Statement

The studies involving human participants were reviewed and approved by UCL Research Ethics Committee (Application 11819/002). A cover letter was provided at the beginning of a survey link, which included consent from participants to participate in this study.

## Author Contributions

SM: conceptualization, methodology design, data curation, data analysis and interpretation of data, and writing—original draft. FS: methodology design, writing review, editing, and supervision. LB: methodology design, writing review, and editing. RH: data curation, writing review, and editing. IB: conceptualization, methodology, data analysis and interpretation, writing review, editing, and supervision. All authors contributed to the article and approved the submitted version.

## Funding

This work was made possible by PhD scholarship and research funding from the Indonesia Endowment Fund for Education (Lembaga Pengelola Dana Pendidikan), Ministry of Finance, Republic of Indonesia (S-295/LPDP.3/2017).

## Conflict of Interest

The authors declare that the research was conducted in the absence of any commercial or financial relationships that could be construed as a potential conflict of interest.

## Publisher's Note

All claims expressed in this article are solely those of the authors and do not necessarily represent those of their affiliated organizations, or those of the publisher, the editors and the reviewers. Any product that may be evaluated in this article, or claim that may be made by its manufacturer, is not guaranteed or endorsed by the publisher.

## Abbreviations

CBET: Competency-based education and training
CPD: Continuing Professional Development
GADF: Global advanced development framework
IADF: Indonesian Advanced Development Framework
IAI: Indonesian Pharmacists Association
LMICs: Low and middle-income countries
MCA: Multiple correspondent analysis
SIG: Special interest group.
