# Supplementary figures and images for: Competency-Based Education: Developing an Advanced Competency Framework for Indonesian Pharmacists
Source: Front Med (Lausanne). 2021 Nov 25;8:769326. doi: 10.3389/fmed.2021.769326 (PMC8655862; doi:10.3389/fmed.2021.769326)

## Supplementary Material

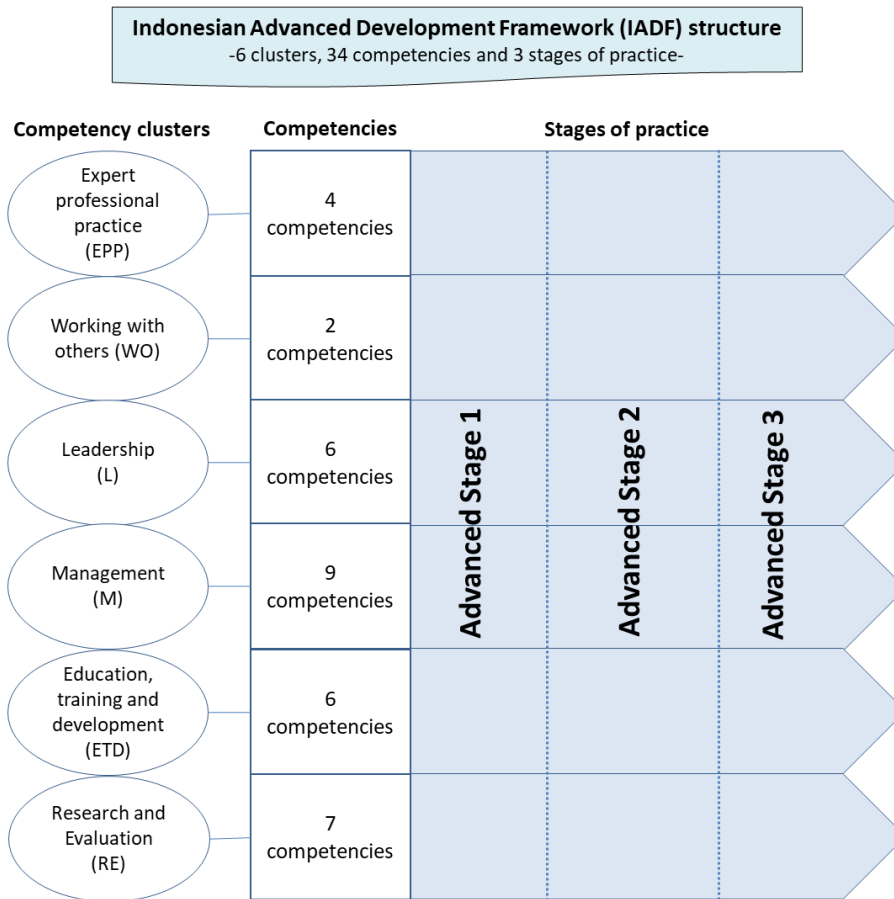

**Indonesian Advanced Development Framework structure**

Supplement: Supplementary file 1 [file Image_1.pdf]
